# lncRNA-803 Suppresses Apoptosis in DF-1 Cells via the miR-6555-3p/MDM4/p53 Axis

**DOI:** 10.3390/genes17040440

**Published:** 2026-04-12

**Authors:** Shuo Han, Jingyi Yang, Yunqiao Qiu, Shuang Zhao, Yingxue Jiang, Liping Han, Limei Han

**Affiliations:** 1 Engineering Laboratory for Tarim Animal Diseases Diagnosis and Control, College of Animal Science and Technology, Tarim University, Alar 843300, China; 120240146@taru.edu.cn (S.H.); ack_emma@163.com (Y.Q.); 2 Key Laboratory of Livestock Infectious Diseases in Northeast China, Ministry of Education, College of Animal Science and Veterinary Medicine, Shenyang Agricultural University, Shenyang 110866, China; yangzhu19950810@163.com (J.Y.); 13842081197@163.com (S.Z.); jiangyingxue1997@163.com (Y.J.); 3 Department of Bioscience, Changchun Normal University, Changchun 130032, China

**Keywords:** apoptosis, lncRNA-803, Marek’s disease, p53 pathway, TP53BP1

## Abstract

Background/Objectives: Long non-coding RNAs (lncRNAs) are integral to the regulation of viral tumorigenesis. We have previously identified that the chicken lncRNA-803, which responds to Marek’s disease virus (MDV), inhibits apoptosis in the chicken embryonic fibroblast cell line DF-1, accompanied by changes in the expression of the p53 protein. Nonetheless, the molecular mechanism of lncRNA-803 in apoptosis has yet to be elucidated. Methods: In this study, through lentivirus-mediated overexpression and knockdown experiments, we determined that the overexpression of lncRNA-803 induces elevated expression levels of murine double minute 2 (*MDM2)*, murine double minute 4 (*MDM4)*, tumor protein p53 (*p53*), and tumor protein p53 binding protein 1 (*TP53BP1)* within the p53 signaling pathway. Results: This modulation subsequently leads to an upregulation of B-cell lymphoma-2 (*Bcl-2)* expression, while concurrently resulting in the downregulation of cysteinyl aspartate specific proteinase 8 (*Caspase-8)*, cysteinyl aspartate specific proteinase 9 (*Caspase-9)*, Bcl-2 associated protein X (*Bax)*, and cysteinyl aspartate specific proteinase 9 (*Caspase-3)* in the apoptosis pathway. In terms of its mechanism, lncRNA-803 functions as a molecular sponge for miR-6555-3p. lncRNA-803 engages in competitive binding with miR-6555-3p, thereby diminishing its inhibitory effect on *MDM4*. Conclusions: These results elucidate that lncRNA-803 modulates apoptosis in DF-1 cells through a novel competing endogenous RNA mechanism involving the miR-6555-3p/*MDM4/p53* axis. These findings provide new insights into the molecular pathogenesis of MDV.

## 1. Introduction

Long non-coding RNAs (lncRNAs) represent a class of non-coding RNAs that regulate gene expression through a variety of mechanisms [1]. LncRNAs are integral elements of the competing endogenous RNA (ceRNA) regulatory network. They are involved in the pathogenesis and progression of various viral tumor diseases [2]. In a previous investigation, we observed a significant upregulation of lncRNA-803 in the spleens of chickens infected with Marek’s disease virus (MDV), and it was found to inhibit apoptosis in DF-1 cells [3]. Marek’s disease (MD) is a viral tumor disease caused by MDV [4]. The extensive application of vaccines has exerted selective pressure on MDV, leading to increased virulence and immune evasion, thereby posing significant challenges for the poultry industry [5]. Investigating the molecular mechanisms underlying MD tumorigenesis from the perspective of non-coding RNA interactions is crucial for the effective prevention and management of MD, as well as for promoting the sustainable development of poultry farming [6].

MD is a transmissible neoplastic disease caused by MDV, characterized by a high incidence rate and challenging control measures. Infected poultry exhibit various symptoms, such as weight loss and diminished egg production, which leads to significant economic losses [7]. MD is consistently recognized as a major threat to the sustainable development of the poultry industry. It also serves as a quintessential animal model for studying herpesvirus-related diseases [8]. The traditional MD vaccines have certain limitations, such as stringent transportation requirements and inadequate prophylactic efficacy. These limitations have prompted international efforts to develop novel genetic engineering vaccines, breed resistant poultry strains, and investigate MDV pathogenesis. These endeavors aim to identify viable targets for the effective prevention and management of MD [9]. Moreover, MD represents a well-established animal model for studying virus-induced lymphoma. It is also noted as the first animal tumor disease for which vaccination has proven preventive [10]. The MD vaccine offers protection for chicks. However, the persistent evolution of MDV’s virulence and its frequent co-infection with other viruses contribute to the widespread dissemination of MD globally [11,12,13].

In contrast to coding regions, the majority of the biological genome is composed of non-coding regions. Recent research has identified a substantial number of non-coding RNAs in both viruses and host cells, primarily including lncRNAs and microRNAs (miRNAs) [2,14]. The expression levels of these functional non-coding RNAs are responsive to the MDV infection process. Therefore, they can serve as molecular markers for the rapid diagnosis of MD. Additionally, they influence the expression levels of other coding genes or non-coding RNAs. Furthermore, lncRNAs function as “sponges” for miRNAs. Through this function, lncRNAs regulate the expression of target genes. This regulation ultimately influences the expression levels and functions of the target messenger RNA [15,16].

LncRNA-803, identified as an MD-associated lncRNA, is derived from chicken spleen tissue. In a prior study, we exposed 1-day-old White Leghorn chicks to MDV and conducted both lncRNA and mRNA sequencing on spleen tissue at 42 days post-infection. Differential expression analysis indicated that lncRNA-803 is responsive to MDV infection, demonstrating increased expression levels in the spleens of infected chickens. Additionally, a significant correlation was identified between the expression of lncRNA-803 and the tumor protein p53 (p53) pathway protein [3]. However, the precise pathway through which lncRNA-803 mediates apoptosis inhibition in DF-1 cells remains to be clarified.

In the present study, we utilized a lentivirus-mediated model to either overexpress or knockdown lncRNA-803 in DF-1 cells. This enabled a detailed investigation into the molecular mechanisms by which lncRNA-803 modulates apoptosis. The expression levels of key proteins within the p53 and apoptosis pathways were evaluated. Furthermore, this research will identify the miRNA interacting with lncRNA-803 and the p53 pathway-related genes targeted by the miRNA. Meanwhile, it will also examine the impact of the miRNA interacting with lncRNA-803 on the apoptosis of DF-1 cells and its underlying regulatory mechanisms. Ultimately, this work aims to establish a ceRNA regulatory network involving lncRNA-803, thereby providing novel insights into the molecular pathogenesis of MDV.

## 2. Materials and Methods

### 2.1. Ethics Statement

Ethical approval was not required for the studies on animals in accordance with the local legislation and institutional requirements because only commercially available established cell lines were used. No human studies are presented in the manuscript.

### 2.2. Cells, Vectors, miRNA

HEK-293T cells and DF-1 cells were purchased from Procell Company (Wuhan, China). A variety of plasmids, including pCDH-MSCV-MCS-EF1-CopGFP-T2A-Puro (hereafter referred to as pCDH), pSIH1-H1-copGFP-T2A-Puro (hereafter referred to as pSIH1), pMD2.G, psPAX2, and pmiR-GLO, were purchased from Miaoling Plasmid Company (Wuhan, China). Several vectors have been constructed for specific applications: the pCDH-lncRNA-803 vector was constructed to synthesize lncRNA-803 overexpression lentivirus, the pSIH1 vector was constructed to synthesize lncRNA-803 knockdown lentivirus, and the pmiR-GLO-lncRNA-803-wild, pmiR-GLO-lncRNA-803-mut, pmiR-GLO-*MDM4*-wild, and pmiR-GLO-*MDM4*-mut vectors were constructed to perform the dual luciferase reporter assay. The chicken miR-6555-3p mimic negative control (NC) and miR-6555-3p mimic were synthesized by Genepharma Company (Suzhou, China).

### 2.3. Establishment of Cell Model

The vectors pCDH, pGreen, pCDH-lncRNA-803, and pGreen-lncRNA-803 were co-transfected with the packaging vectors pMD.2G and psPAX2 into HEK 293T cells. These cells were maintained at 37 °C in an atmosphere containing 5% CO_2_ for a duration of 72 h to facilitate routine cultivation. Subsequently, the supernatants containing lentiviruses for either overexpressed or knocked down lncRNA-803 were collected. Transfection was conducted utilizing the PEI 40K reagent (G1802, Servicebio, Wuhan, China). Following the collection of viral suspensions, the viral suspension was concentrated using the lentivirus concentration kit (G1801, Servicebio, Wuhan, China). The titer of the concentrated viral suspension was quantitatively assessed using the lentivirus titer detection kit (G1804, Servicebio, Wuhan, China). After that, the viral suspensions were stored at −80 °C. For the construction of a stable cell model, DF-1 cells were seeded at a density of 5 × 10^5^ cells per well in 6-well plates and cultured for 24 h. Subsequently, the medium was replaced with fresh opti-MEM medium. Lentivirus suspensions, at a final concentration of 1 × 10^11^ TU/mL and supplemented with 8 μg/mL polybrene, were added to the opti-MEM medium. The DF-1 cells were further cultured for an additional 72 h to establish the cell models with overexpressed or knocked down lncRNA-803.

### 2.4. Real-Time Quantitative Polymerase Chain Reaction (RT-qPCR)

The total RNA was extracted from DF-1 cells infected with pCDH, pGreen, pCDH-lncRNA-803, and pGreen-lncRNA-803 lentiviruses using RNAiso (DP430, Tiangen, Beijing, China). cDNA synthesis was conducted utilizing the HiScript III RT SuperMix for qPCR (+gDNA wiper) (R323, Vazyme, Nanjing, China). The expression levels of genes and lncRNA-803 were quantified using ChamQ SYBR qPCR Master Mix (Q311, Vazyme, Nanjing, China). For miRNA analysis, reverse transcription was performed using the miRNA First Strand cDNA Synthesis Kit (Tailing Reaction) (B532451, Sangon, Shanghai, China), followed by RT-qPCR using the miRNAs qPCR Kit (B532461, Sangon, Shanghai, China). The relative expression levels of lncRNA-803, genes, and miRNAs were calculated using the 2^−ΔΔCt^ method. The results are presented as relative fold changes in the value of the control group, normalized to the endogenous controls GAPDH or U6. The primer sequences employed for RT-qPCR are detailed in Table S1.

### 2.5. Flow Cytometry

The apoptosis rate in DF-1 cells was assessed using the Annexin V-APC/DAPI Apoptosis Detection Kit (E-CK-A258, Elabscience, Wuhan, China). Initially, DF-1 cells were washed three times with PBS, followed by digestion with 0.25% trypsin for 2 min. Subsequently, the DF-1 cells were collected in DMEM supplemented with 10% foetal bovine serum, and centrifuged at 300× *g* for 5 min. A total of 5 × 10^5^ digested DF-1 cells were then washed twice with PBS, centrifuged again at 300× *g* for 5 min, and resuspended in 500 μL of diluted 1 × Annexin V binding buffer. Next, the cells were incubated with 5 μL of Annexin V-APC reagent and 5 μL of DAPI reagent in the dark environment for 20 min at room temperature. The detection was performed using a BD FACSAria III flow cytometer (Becton, Dickinson and Company, Franklin, MA, USA).

### 2.6. Terminal Deoxynucleotidyl Transferase-Mediated dUTP Nick End Labeling (TUNEL) Assay

The TUNEL assay was performed using the One-step TUNEL In Situ Apoptosis Kit (Red, Elab Fluor 594) (E-CK-A322, Elabscience, Wuhan, China). The DF-1 cells were washed twice with PBS and subsequently fixed in 4% paraformaldehyde for 30 min at 37 °C. Next, the DF-1 cells were incubated with 0.2% Triton X-100 solution for 10 min at 37 °C. The cells were incubated with 100 μL of TdT equilibration buffer at 37 °C for 30 min, followed by incubation with 50 μL of staining solution for 2 h at 37 °C in a dark environment. Finally, the cells were treated with DAPI reagent for 5 min at room temperature. The fluorescence intensity was assessed using an inverted microscope (DM4000B, Leica, Wetzlar, Germany).

### 2.7. Dual Luciferase Reporter Assay

A dual luciferase reporter assay was conducted to elucidate the interactions between lncRNA-803 and miR-6555-3p, as well as the interaction between miR-6555-3p and *MDM4*. The DF-1 cells were transfected with 2 μg of either the pmiRGLO, pmiRGLO-wild, or pmiRGLO-mut vectors, along with 60 pmol of either the miR-6555-3p mimic NC or the miR-6555-3p mimic using Lipofectamine 2000 (11668030, Thermofisher, Waltham, MA, USA). The fluorescence activity in DF-1 cells was quantified using a dual luciferase reporter assay kit (DL101-01, Vazyme, Nanjing, China) in accordance with the manufacturer’s instructions.

### 2.8. Western Blot

Western blot analysis was performed following standard protocols, using antibodies specific to p53 (BF8013, Affinity, Changzhou, China), tumor protein p53 binding protein 1 (TP53BP1) (AB175933, Abcam, Cambridge, MA, USA), murine double minute 2 (MDM2) (TB3975, Abmart, Shanghai, China), murine double minute 4 (MDM4) (TD7532, Abmart, Shanghai, China), Bcl-2 associated protein X (Bax) (50599-2-Ig, Proteintech, Wuhan, China), B-cell lymphoma-2 (Bcl-2) (26593-1-AP, Proteintech, Wuhan, China), cysteinyl aspartate specific proteinase 8 (Caspase-8) (YT6191, Immunoway, Suzhou, China), cysteinyl aspartate specific proteinase 9 (Caspase-9) (YM8173, Immunoway, Suzhou, China), cysteinyl aspartate specific proteinase 3 (Caspase-3) (T55501 Abmart, Shanghai, China), β-actin (P30002, Abmart, Shanghai, China) and horseradish peroxidase-labeled goat anti-rabbit IgG (D110058, Sangon, Shanghai, China). The bands were visualized using an enhanced chemiluminescence detection system (P0018S, Beyotime, Shanghai, China). ImageJ2 software (National Institutes of Health, Bethesda, MD, USA) was employed to quantify the gray value of protein bands.

### 2.9. Bioinformatics Analysis

The online platforms LncTar (https://www.cuilab.cn/lnctar accessed on 13 March 2023), miRDB (https://mirdb.org/ accessed on 13 March 2023), and TargetScan (https://www.targetscan.org/vert_80/ accessed on 21 June 2023) were utilized to predict the miRNAs targeted by lncRNA-803, as well as the potential target genes of these miRNAs. Subsequently, the Database for Annotation, Visualization, and Integrated Discovery (https://davidbioinformatics.nih.gov/ accessed on 21 June 2023) was employed to predict and analyze the signaling pathways associated with these target genes.

### 2.10. Statistical Analysis

All the experiments were performed with three independent biological replicates (*n* = 3). Each biological replicate was measured in three technical replicates. Statistical comparisons between different groups were conducted using the independent sample *t*-test in SPSS 19.0 (IBM, Armonk, NY, USA), with data presented as mean ± standard deviation. Differences were considered statistically significant if *p* < 0.05.

## 3. Results

### 3.1. lncRNA-803 Suppresses Apoptosis of DF-1 Cells

**Figure 1:**
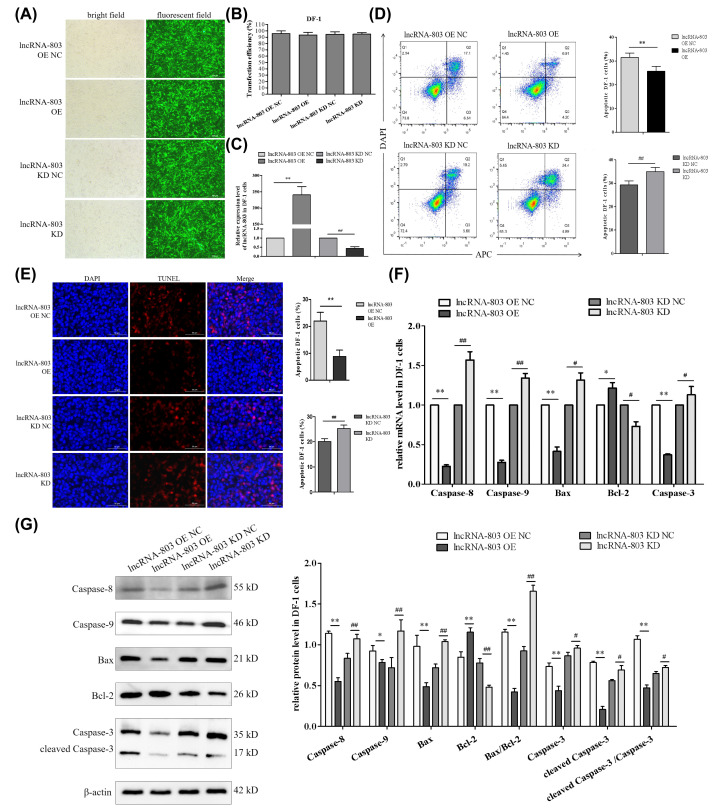
The effect of lncRNA-803 on the apoptosis pathway of DF-1 cells. (**A**) DF-1 cells were infected with lentivirus to overexpress or knockdown lncRNA-803. (**B**) Statistical analysis of the infection efficiency of lentiviruses engineered to overexpress or knockdown lncRNA-803. (**C**) Relative expression levels of lncRNA-803 in DF-1 cells. (**D**) Effect of lncRNA-803 on the middle and late apoptosis rates of DF-1 cells. (**E**) Effect of lncRNA-803 on the late apoptosis rate of DF-1 cells. (**F**) Effect of lncRNA-803 on the mRNA expression levels of genes involved in the apoptosis pathway in DF-1 cells. (**G**) Effect of lncRNA-803 on the expression of apoptosis pathway-related genes in DF-1 cells. * represents a significant difference compared with lncRNA-803 overexpression control group. ** represents an extremely significant difference compared with lncRNA-803 overexpression control group. # represents a significant difference compared with lncRNA-803 knockdown control group. ## represents an extremely significant difference compared with lncRNA-803 knockdown control group. Ns means the difference is not significant. (*n* = 3).

To explore the molecular mechanism by which lncRNA-803 regulates apoptosis in DF-1 cells, the cell models with overexpression and knockdown of lncRNA-803 were established. Lentiviruses engineered to overexpress or knockdown lncRNA-803 were inoculated into DF-1 cells. Seventy-two hours post-inoculation, the DF-1 cells in different groups all exhibited strong green fluorescence (Figure 1A), with the proportion of green fluorescent cells exceeding 95% (Figure 1B). Relative to the control group, the expression levels of lncRNA-803 were significantly increased or decreased in DF-1 cells following overexpression or knockdown, respectively (Figure 1C). The apoptosis rate in DF-1 cells overexpressing lncRNA-803 was significantly reduced, whereas the apoptosis rate was significantly increased in cells with lncRNA-803 knockdown (Figure 1D). In comparison to the control group, the overexpression of lncRNA-803 significantly upregulated the relative expression levels of *Bcl-2* mRNA and protein. Conversely, there was a significant or extremely significant downregulation in the relative expression levels of *Caspase-8*, *Caspase-9*, *Bax*, and *Caspase-3* mRNA and protein. Additionally, the protein expression levels of Bax/Bcl-2, cleaved-Caspase-3, and the cleaved-Caspase-3/Caspase-3 ratio were markedly decreased. Upon knockdown of lncRNA-803, the expression levels of these genes exhibited an inverse trend compared to the overexpression of lncRNA-803 (Figure 1E,F).

### 3.2. The Nucleotide Positions 85–92 of lncRNA-803 Interact with miR-6555-3p

**Figure 2:**
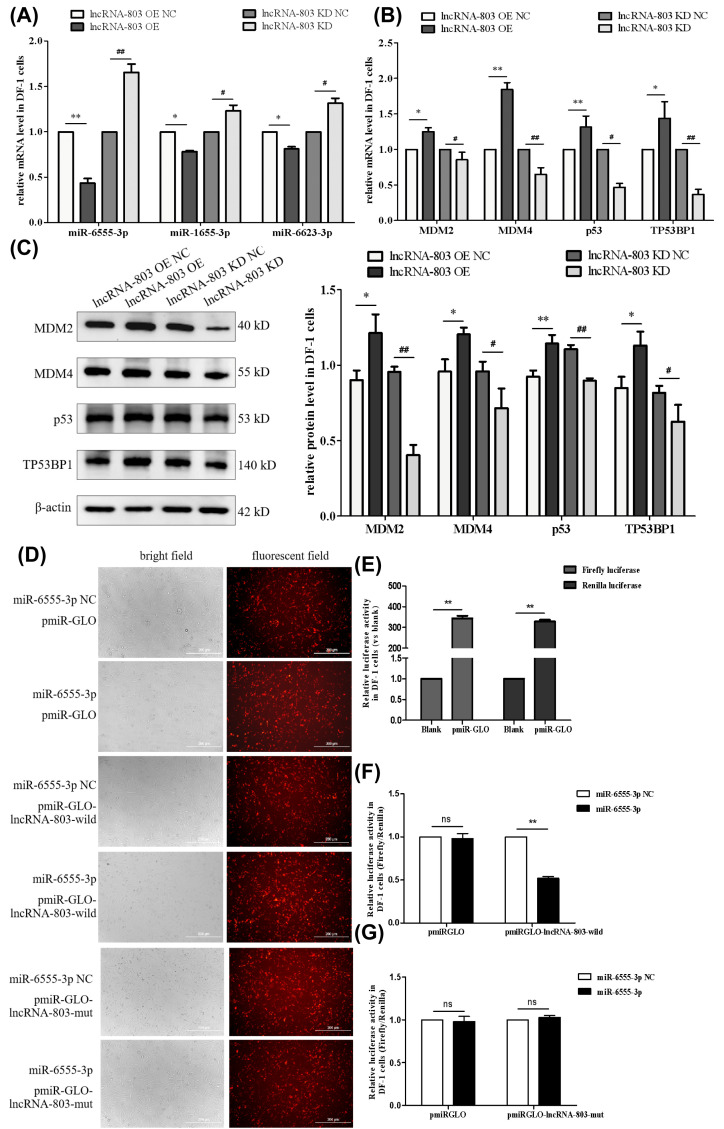
Identification of miRNAs related to the p53 pathway that interact with lncRNA-803. (**A**) Effect of lncRNA-803 on the mRNA level of genes of tomor protein p53 (p53) pathway in DF-1 cells. (**B**) Effect of lncRNA-803 on the protein level of genes of p53 pathway in DF-1 cells. (**C**) Effect of lncRNA-803 on miRNAs expression in DF-1 cells. * represents significant difference compared with lncRNA-803 overexpression control group. ** represents an extremely significant difference compared with lncRNA-803 overexpression control group. # represents significant difference compared with lncRNA-803 knockdown control group. ## represents an extremely significant difference compared with lncRNA-803 knockdown control group. ns represents no significant difference. (**D**) DF-1 cells co-transfected with miR-6555-3p mimics and dual luciferase reporter vector. (**E**) Effect of pmiR-GLO transfection on firefly luciferase and renilla luciferase activities in DF-1 cells. (**F**) The effect of co-transfection with dual luciferase reporter vector and miR-6555-3p mimics on relative luciferase activity in DF-1 cells. (**G**) DF-1 cells co-transfected with miR-6555-3p mimics and dual luciferase reporter vector. * represents a significant difference. ** represents an extremely significant difference. (*n* = 3).

LncRNAs can bind to miRNAs and regulate the functions of related target genes through the ceRNA mechanism. To explore the interacting miRNAs of lncRNA-803, a bioinformatics prediction analysis was conducted. Bioinformatics analysis predicted potential interacting miRNAs of lncRNA-803, including miR-6555-3p, miR-1655-5p, and miR-6623-3p. The target genes of these miRNAs, such as *MDM2* and *MDM4*, were enriched in the *p53* pathway. To preliminarily validate the bioinformatics predictions, the expression levels of these miRNAs were quantified. The results revealed that in DF-1 cells overexpressing lncRNA-803, the expression level of miR-6555-3p was extremely significantly decreased, while the expression levels of miR-1655-5p and miR-6623-3p were significantly reduced. The knockdown of lncRNA-803 led to an extremely significant upregulation in the expression level of miR-6555-3p, alongside a significant increase in the expression levels of miR-1655-5p and miR-6623-3p (Figure 2A). Therefore, we initially selected miR-6555-3p as the candidate interacting miRNA for lncRNA-803.

The bioinformatics analysis predicted that miR-6555-3p might directly bind to the 3′-untranslated region (UTR) of *MDM4*. This interaction is hypothesized to modulate the p53 signaling pathway. Consequently, the expression levels of key genes within the p53 pathway were assessed. The findings indicate that the overexpression of lncRNA-803 resulted in significant or extremely significant increases in the mRNA and protein expression levels of *MDM2*, *MDM4*, *p53*, and *TP53BP1*. Conversely, the knockdown of lncRNA-803 led to significant or extremely significant reductions in the mRNA and protein expression levels of these genes (Figure 2B,C).

To verify the interaction between lncRNA-803 and miR-6555-3p, we conducted a dual luciferase reporter assay. In DF-1 cells, co-transfection with pmiR-GLO-lncRNA-803-wild and miR-6555-3p mimics significantly reduced the luciferase activity compared to cells transfected with miR-6555-3p NC, indicating an interaction between lncRNA-803 and miR-6555-3p (Figure 2D–F). However, when nucleotides 85−92 of lncRNA-803 were mutated, co-transfection with miR-6555-3p did not alter luciferase activity, suggesting that miR-6555-3p specifically binds to nucleotides 85−92 of lncRNA-803 (Figure 2G).

### 3.3. miR-6555-3p Promotes Apoptosis in DF-1 Cells

**Figure 3:**
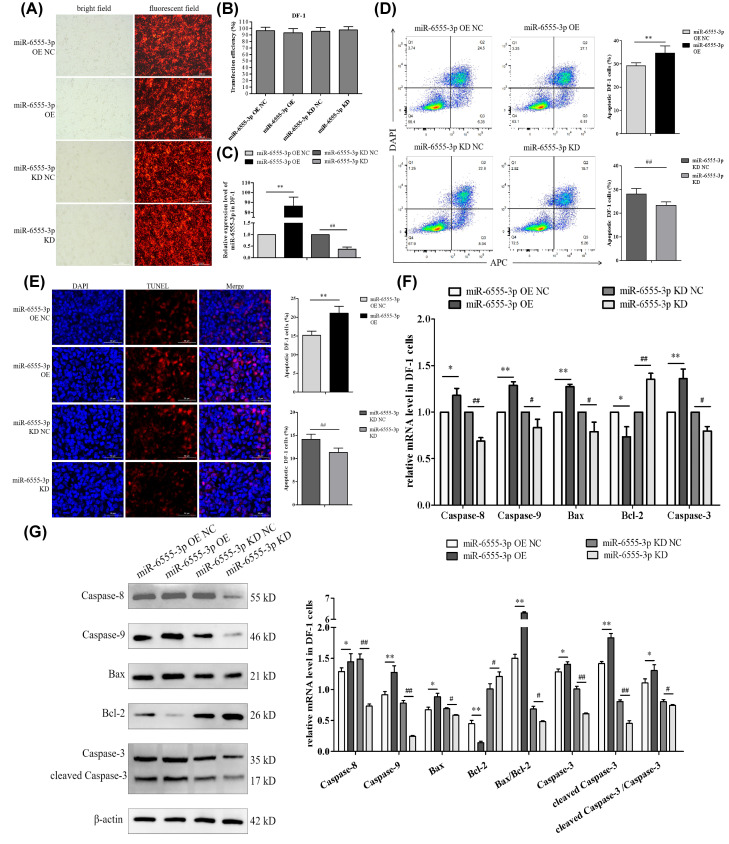
The effect of miR-6555-3p on the apoptosis pathway of DF-1 cells. (**A**) DF-1 cells transfected with miR-6555-3p mimic or inhibitor. (**B**) Statistical results on the transfection efficiency of miR-6555-3p mimic or inhibitor. (**C**) The relative expression of miR-6555-3p in DF-1 cells with miR-6555-3p overexpression or knockdown. (**D**) Effect of miR-6555-3p on middle and late apoptosis rate of DF-1 cells. (**E**) Effect of miR-6555-3p on late apoptosis rate of DF-1 cells. (**F**) Effect of miR-6555-3p on the mRNA level of genes of apoptosis pathway in DF-1 cells. (**G**) Effect of miR-6555-3p on the protein levels of genes of apoptosis pathway in DF-1 cells. * represents significant difference compared with miR-6555-3p overexpression control group. ** represents an extremely significant difference compared with miR-6555-3p overexpression control group. # represents significant difference compared with miR-6555-3p knockdown control group. ## represents an extremely significant difference compared with miR-6555-3p knockdown control group. (*n* = 3).

Although the interaction between lncRNA-803 and miR-6555-3p has been identified, it remains unknown whether miR-6555-3p regulates apoptosis in DF-1 cells. Therefore, DF-1 cell models with either overexpression or knockdown of miR-6555-3p were established. Following a 48 h transfection with miR-6555-3p mimics and inhibitors, the proportion of green fluorescent DF-1 cells in each group exceeded 90% (Figure 3A,B), confirming the successful transfection. Relative to the control group, the expression levels of miR-6555-3p were markedly upregulated or downregulated in DF-1 cells transfected with miR-6555-3p mimics or inhibitors (Figure 3C), demonstrating the successful establishment of miR-6555-3p overexpression and knockdown in DF-1 cells. The overexpression of miR-6555-3p resulted in an extremely significant increase in the apoptosis rate of DF-1 cells, whereas its knockdown led to an extremely significant decrease in apoptosis (Figure 3D,E), suggesting that miR-6555-3p facilitates apoptosis. In DF-1 cells with miR-6555-3p overexpression, the relative expression levels of *Bcl-2* mRNA and protein were significantly or extremely significantly reduced, while the expression levels of *Caspase-8*, *Caspase-9*, *Bax*, and *Caspase-3* were significantly or extremely significantly elevated. The expression levels of Bax/Bcl-2, cleaved-Caspase-3, and the cleaved-Caspase-3/Caspase-3 ratio were significantly or extremely significantly elevated. After miR-6555-3p was knocked down, the expression levels of these apoptosis-related genes exhibited an inverse trend compared to cells with miR-6555-3p overexpression (Figure 3F,G).

### 3.4. miR-6555-3p Interacts with the 3′-UTR of MDM4

**Figure 4:**
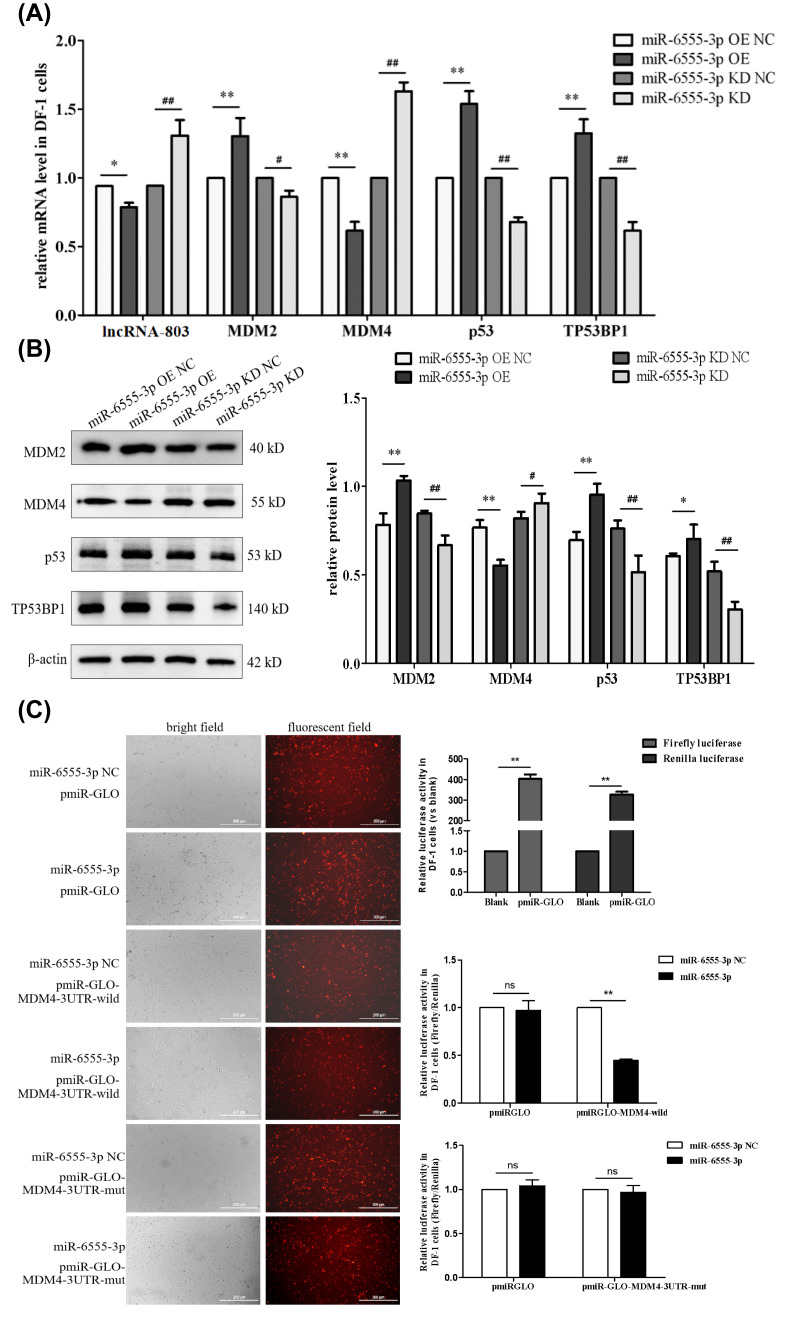
Identification of murine double minute 4 (*MDM4*) related to the p53 pathway that interact with miR-6555-3p. (**A**) Effect of miR-6555-3p on the mRNA level of genes of p53 pathway in DF-1 cells. (**B**) Effect of miR-6555-3p on the protein level of genes of p53 pathway in DF-1 cells. * represents significant difference compared with lncRNA-803 overexpression control group. ** represents an extremely significant difference compared with lncRNA-803 overexpression control group. # represents significant difference compared with lncRNA-803 knockdown control group. ## represents an extremely significant difference compared with lncRNA-803 knockdown control group. ns represents no significant difference. (**C**) DF-1 cells co-transfected with miR-6555-3p and dual luciferase reporter vector. * represents a significant difference. ** represents an extremely significant difference. (*n* = 3).

*MDM4* is a candidate target gene of miR-6555-3p within the p53 pathway. Therefore, the expression levels of key genes in the p53 pathway were evaluated. In DF-1 cells, the overexpression of miR-6555-3p resulted in significant or extremely significant increases in the relative mRNA and protein expression levels of *MDM2*, *p53*, and *TP53BP1*. Conversely, the relative mRNA and protein expression levels of lncRNA-803 and *MDM4* were significantly or extremely significantly reduced. Conversely, the knockdown of miR-6555-3p led to significant or extremely significant reductions in the relative mRNA and protein expression levels of *MDM2*, *p53*, and *TP53BP1*, while the relative mRNA and protein expression levels of lncRNA-803 and *MDM4* significantly increased (Figure 4A,B).

To determine the interaction relationship and binding site between miR-6555-3p and MDM4, the dual luciferase reporter assay was conducted. In comparison to the co-transfection group involving pmiR-GLO-*MDM4*-3′-UTR-wild and miR-6555-3p NC, the relative luciferase activity in DF-1 cells co-transfected with pmiR-GLO-*MDM4*-3′-UTR-wild and miR-6555-3p mimics was significantly diminished. This suggests an interaction between miR-6555-3p and *MDM4* in DF-1 cells. Upon successful transfection of miR-6555-3p mimics and pmiR-GLO into DF-1 cells, no significant difference in relative luciferase activity was observed between DF-1 cells co-transfected with pmiR-GLO-*MDM4*-3′-UTR-mut and miR-6555-3p mimics, compared to those co-transfected with pmiR-GLO-*MDM4*-3′-UTR-mut and miR-6555-3p mimics NC (Figure 4C). MiR-6555-3p specifically interacts with nucleotides 3032−3037 of the *MDM4*-3′-UTR in DF-1 cells.

## 4. Discussion

MD is characterized as a proliferative infectious disease of lymphoid tissues [17]. Due to the significant challenges associated with its prevention and control, MD is consistently recognized as a principal disease posing a threat to the sustainable development of the poultry industry [18]. Researchers have identified several lncRNAs associated with MD, including lincRNA-satb1 [19], lincRNA-GALMD3 [20], and lincRNA-GALMD1 [21]. In previous work, we successfully developed a lncRNA-mRNA co-expression network for MDV infection in chicken spleen tissues, identifying lncRNA-803 and lncRNA-9802 as being associated with *TP53BP1* [3,22]. Notably, lncRNA-803 has been demonstrated to inhibit apoptosis in DF-1 cells. The target genes of lncRNA-803 were significantly enriched in the *p53* and apoptosis pathways [3]. Nevertheless, further investigation is required to determine whether lncRNA-803 regulates apoptosis via the *p53* pathway.

In the preliminary work, the lipofectamine 2000 reagent was employed to transfect the pEGFP-C1-lncRNA-803 and pGreen-lncRNA-803-shRNA vectors, thereby successfully establishing DF-1 cell models with either overexpression or knockdown of lncRNA-803 [23]. Nevertheless, the transfection efficiency required enhancement. In the current study, a three-plasmid system was utilized to generate lentiviruses in HEK-293T. This system comprised the encapsulating plasmid pMD2.G, the packaging plasmid psPAX2, and the transfer plasmid pCDH-lncRNA-803 (or pSIH1-lncRNA-803-shRNA) [24,25]. Following infection with lentiviruses that overexpressed or knocked down lncRNA-803 for 72 h, it was observed that the infection efficiency exceeded 95% in DF-1 cells. This demonstrates the successful establishment of cell lines for both the overexpression and knockdown of lncRNA-803.

Building upon the successful establishment of lncRNA-803 overexpression and knockdown cell models, this study utilized flow cytometry and TUNEL assays to assess the apoptosis rate in DF-1 cells. Consistent with previous findings, lncRNA-803 inhibits apoptosis in DF-1 cells [3]. The process of apoptosis involves many associated proteins, with Bax, Bcl-2, and Caspase-3 serving as key effectors [26,27]. The activation of Caspase-8 and Caspase-9 is known to initiate the activation of Caspase-3 [28,29]. Caspase-3 subsequently processes Caspase-9 into a p10 fragment that lacks the XIAP docking domain, thereby further facilitating apoptosis [30]. In DF-1 cells, the overexpression of lncRNA-803 resulted in an upregulation of *Bcl-2* expression, while the expression levels of *Caspase-8*, *Caspase-9*, *Bax*, and *Caspase-3* were downregulated. The changes in the expression of these genes were consistent with the decreased apoptosis rate of DF-1 cells that lncRNA-803 was overexpressed. These results indicate that lncRNA-803 inhibits apoptosis in DF-1 cells by promoting the expression levels of *Bcl-2* and reducing those of *Caspase-8*, *Caspase-9*, *Bax*, and *Caspase-3*.

It is widely recognized that Bax, Caspase-8, Caspase-9, and Caspase-3 are key pro-apoptotic proteins, while Bcl-2 is a pivotal anti-apoptotic protein [31]. Changes in Bax, Bcl-2, Caspase-8, and Caspase-9 expression can activate Caspase-3, thereby impacting the ultimate execution of the apoptotic process [32]. LncRNA-803 inhibits apoptosis in DF-1 cells by down-regulating pro-apoptotic proteins and up-regulating anti-apoptotic proteins. The precursor form of Caspase-3 undergoes cleavage to generate cleaved-Caspase-3, which is essential for its pro-apoptotic function [33]. Consequently, the elevation in the levels of Bax/Bcl-2, activated cleaved-Caspase-3, and the ratio of cleaved-Caspase-3/Caspase-3 is frequently considered as a critical effector in promoting apoptosis [34,35]. In light of this, the expression levels of Bax/Bcl-2, activated cleaved-Caspase-3, and cleaved-Caspase-3/Caspase-3 proteins were examined [36,37]. We found that lncRNA-803 inhibits apoptosis by promoting the expression levels of Bcl-2 and reducing those of Caspase-8, Caspase-9, Bax, and Caspase-3. The expression levels of these proteins align with the finding that the apoptosis rate in DF-1 cells decreased.

Our previous research has demonstrated a correlation between lncRNA-803 and TP53BP1. TP53BP1 serves as a co-regulator of the tumor suppressor protein p53, which functions as a sensor for damage repair [38]. Both TP53BP1 and p53 are involved in the regulation of cell cycle arrest and apoptosis in cells experiencing DNA damage, thereby playing a vital role in maintaining genomic stability and preventing cancer development [39,40,41]. In earlier investigation, we conducted functional gain-of-function and loss-of-function analyses of lncRNA-803 in DF-1 cells. Our findings revealed that the expression levels of p53 and TP53BP1 in DF-1 cells exhibited consistent and systematic changes in response to the overexpression or knockdown of lncRNA-803 [3]. This implies that the *p53* signaling pathway may be integral to the regulation of apoptosis by lncRNA-803. Under normal physiological conditions, the expression of the p53 protein is maintained at a relatively low level due to the inhibitory effects of its negative regulatory factors, MDM2 and MDM4 [42,43]. Upon the transmission of external stimuli into the cell, the regulatory interactions among MDM2, MDM4, and p53 are modified, resulting in changes in the activity of the *p53* pathway [44]. Furthermore, the Meq protein encoded by MDV has been demonstrated to directly interact with the *p53* protein [45]. The exogenous expression of Meq leads to the suppression of *p53*-mediated gene transcriptional activity and apoptosis [45]. Investigating the potential modulation of apoptosis by lncRNA-803 via the *p53* pathway is crucial for elucidating the host–virus interaction.

To delineate the molecular mechanisms through which lncRNA-803 regulates apoptosis, an investigation was conducted into the transcriptional and translational expression levels of genes within the p53 signaling pathway, specifically *MDM2*, *MDM4*, *TP53BP1*, and *p53*. In DF-1 cells, the overexpression of lncRNA-803 leads to an upregulation of *MDM4* and *p53*. Additionally, lncRNA-803 overexpression enhances the expressions of *MDM2, MDM4, p53* and *TP53BP1* in DF-1 cells. MDM2 and MDM4 are recognized as the primary negative regulators of p53, binding to p53 to form a stable complex that modulates its activity, primarily through the ubiquitination-mediated degradation of the p53 protein [46]. The *MDM2*-*p53* and *MDM4-p53* axis demonstrates diverse regulatory functions across various physiological and pathological contexts [47,48].

The ceRNA mechanism is a crucial mode of interaction between coding and non-coding genes in biological systems [49]. LncRNAs within this network can bind to miRNAs at competitive manner, functioning as “sponges” and thereby influencing various biological processes [50]. To elucidate the molecular mechanism by which lncRNA-803 regulates apoptosis via the *p53* pathway, focusing on non-coding RNA interactions, the miRNAs that interact with lncRNA-803 were predicted. It was found that lncRNA-803 potentially interacts with miR-6555-3p, miR-1655-5p, and miR-6623-3p, thereby modulating the expression of *MDM2* and *MDM4*, ultimately impacting the activity of the *p53* pathway. The dual luciferase reporter system is a widely recognized technique for detecting interactions between non-coding RNAs [51,52]. This research employed the dual luciferase reporter assay to elucidate the interaction dynamics and specific binding sites between lncRNA-803 and miR-6555-3p in DF-1 cells. The nucleotide positions 85−92 of lncRNA-803 were found to interact with miR-1655-5p. Despite identifying miR-6555-3p as the interacting miRNA of lncRNA-803, further investigation is required to determine its role in regulating apoptosis.

miR-6555-3p, a miRNA identified in chicken embryos, has not yet been functionally described [53]. Successful establishment of cell lines with overexpressed and knocked down miR-6555-3p was achieved after transfection with miR-6555-3p mimics and inhibitors. This study marks the first successful establishment of a cell model with either overexpressed or knocked down miR-6555-3p, providing a foundation for further functional analyses. Subsequent investigations have demonstrated that miR-6555-3p facilitates apoptosis in DF-1 cells. This study is the first to elucidate the function of miR-6555-3p, thereby corroborating the interaction between lncRNA-803 and miR-6555-3p. This further confirms miR-6555-3p as a critical interaction factor for lncRNA-803 in apoptosis regulation.

The study assessed gene expression levels related to the *p53* and apoptosis pathways to investigate whether miR-6555-3p modulates the *p53* pathway and mediates lncRNA-803′s regulatory effects on apoptosis. The overexpression of miR-6555-3p resulted in higher levels of *p53*, *MDM2*, and *TP53BP1*, while *MDM4* levels decreased. p53 is a principal inducer of the apoptosis pathway [54], and it interacts with several Bcl-2 family members [55]. The overexpression of miR-6555-3p exhibited elevated levels of Caspase-8, Caspase-9, Bax, Caspase-3, and cleaved-Caspase-3, while reducing Bcl-2 expression. This trend of change is consistent with the result that miR-6555-3p promotes apoptosis in DF-1 cells. The above research results indicate that miR-6555-3p promotes apoptosis in DF-1 cells and exerts a function opposite to that of lncRNA-803.

The lncRNA-mediated ceRNA regulatory network is pivotal in the regulation of apoptosis. Bioinformatics prediction analysis indicated that miR-6555-3p may target the 3′-UTR of *MDM4*. This study demonstrated that miR-6555-3p in DF-1 cells can directly interact with the 3′-UTR of *MDM4*, specifically at the nucleotide positions 3032−3037. The 3′-UTR of mRNA is integral to the regulation of mRNA stability, translation, and localization [56]. MDM4 serves as a negative regulator of the p53 protein [57]. This finding elucidates the mechanism by which the overexpression of lncRNA-803 leads to a reduction in the expression level of miR-6555-3p, concurrently resulting in an upregulation of MDM4 expression. We have now described a complete ceRNA regulatory axis in which lncRNA-803 targets miR-6555-3p, thus removing the suppressive impact of miR-6555-3p on *MDM4*.

MDM2 and MDM4 are well-established negative regulators of the p53 protein [58]. Notably, our findings demonstrate that the overexpression of lncRNA-803 leads to the simultaneous upregulation of *MDM2*, *MDM4*, and *p53*, a result that may initially seem paradoxical. However, this observation highlights the complexity of the *p53* regulatory network. The promoter region of *MDM2* contains the *p53* response elements, enabling transcriptional upregulation of *MDM2* following *p53* activation, thereby establishing a classic negative feedback loop [59]. Thus, the observed concurrent upregulation of *MDM2* and *p53* suggests the proper functioning of this feedback mechanism. Upon lncRNA-803 overexpression, the p53 protein level increases, promoting its binding to the *MDM2* promoter region and subsequently activating *MDM2* transcription, resulting in elevated MDM2 protein levels. Furthermore, *MDM4* is the target of miR-6555-3p. lncRNA-803 functions as a molecular sponge for miR-6555-3p, leading to a decrease in miR-6555-3p expression levels. This decrease alleviates the inhibitory effect of miR-6555-3p on *MDM4*, thereby enhancing *MDM4* stability, as evidenced by a direct upregulation of the MDM4 protein.

In summary, this study reveals that lncRNA-803 regulates apoptosis in DF-1 cells through the ceRNA mechanism. lncRNA-803 functions as a molecular sponge for miR-6555-3p, thereby competitively binding to miR-6555-3p and alleviating its suppression of the target gene *MDM4*. Specifically, lncRNA-803 acts as a molecular sponge for miR-6555-3p, competing with it and reducing its inhibition of the target gene *MDM4.* Following this interaction, the expression of genes associated with the *p53* and apoptosis pathways is influenced, resulting in the inhibition of apoptosis in DF-1 cells. This research uncovers a novel regulatory pathway mediated by the lncRNA-803/miR-6555-3p/*MDM4/p53* axis, offering a theoretical foundation for further understanding the MDV pathogenesis.

## Data Availability

The original contributions presented in this study are included in the article/Supplementary Material. Further inquiries can be directed to the corresponding authors.

## Supplementary Materials

The following supporting information can be downloaded at: https://www.mdpi.com/article/10.3390/genes17040440/s1, Table S1 Primer sequences for RT-qPCR.
